# Profiling 92 circulating neurobiological proteins identifies novel candidate biomarkers of long-term cognitive outcome after ischemic stroke

**DOI:** 10.1038/s41598-025-99735-w

**Published:** 2025-05-02

**Authors:** Cecilia Lagging, Annie Pedersen, Max Petzold, Sofia Furutjäll, Hans Samuelsson, Katarina Jood, Tara M. Stanne, Christina Jern

**Affiliations:** 1 Institute of Biomedicine, Department of Laboratory Medicine, the Sahlgrenska Academy, University of Gothenburg, Box 440, 405 30 Gothenburg, Sweden; 2 Region Västra Götaland, Sahlgrenska University Hospital, Department of Clinical Genetics and Genomics, Gothenburg, Sweden; 3 School of Public Health and Community Medicine, Institute of Medicine, the Sahlgrenska Academy, University of Gothenburg, Gothenburg, Sweden; 4 Institute of Neuroscience and Physiology, Department of Clinical Neuroscience, the Sahlgrenska Academy, University of Gothenburg, Gothenburg, Sweden; 5 Region Västra Götaland, Sahlgrenska University Hospital, Department of Neurology, Gothenburg, Sweden; 6 Department of Psychology, Faculty of Social Sciences, University of Gothenburg, Gothenburg, Sweden

**Keywords:** Ischemic stroke, Outcome, Cognition, Biomarkers, Proteins, Stroke, Proteomics

## Abstract

The biological underpinnings of post-stroke cognitive function are largely unknown, and protein investigations can point towards important pathways for further study. We profiled plasma levels of 91 neurology-related proteins (Olink Neurology panel) and serum Neurofilament light chain (NfL) levels in 205 cases in the Sahlgrenska Academy Study on Ischemic Stroke. Blood was sampled in the acute and convalescent (3 months post-stroke) phase. Cognitive outcome was evaluated by the Barrow Neurological Institute Screen for Higher Cerebral Functions 7 years post-stroke. In linear regression models, 6 and 5 proteins in the acute and convalescent phase, respectively, were univariably associated with cognitive outcome at False Discovery Rate (FDR) < 0.05, and 9 and 8 at *p* < 0.05 after adjustment for age, sex, education and sampling day (model 1) and/or additional adjustment for stroke severity (model 2). Of these, 15 proteins contributed with information in multi-protein models on at least one time-point. These included brain-expressed proteoglycans (NCAN, BCAN, GPC5, SPOCK1); contactin-5 (CNTN5); metabolic enzymes (HAGH, NMNAT1); cluster of differentiation (CD)-proteins (SIGLEC1, CLEC10A, CD200R1); GDNF family receptor alpha-1 (GFR-alpha-1); brorin (VWC2); beta-nerve growth factor (beta-NGF); myostatin (GDF-8); and NfL. We identified novel candidate protein biomarkers of post-stroke cognitive outcome that likely reflect different biological processes, warranting further exploration.

**Supplementary Information:**

The online version contains supplementary material available at 10.1038/s41598-025-99735-w.

## Introduction

The incidence of ischemic stroke before 70 years of age is increasing^1^. Globally, about 60% of the people who are currently living with the consequences of ischemic stroke are under the age of 70 years^2^, and they face the risk of living with these consequences for many years. Despite that a large proportion of patients in this age group has mild stroke as defined by the degree of neurological impairment, results from recent studies highlight that unfavorable outcomes are common. These include cognitive impairment^3,4^ as well as fatigue, depressive symptoms^5,6^ and a relatively high risk of recurrent vascular events^7–10^. We and others have shown that reduced cognitive function in ischemic stroke survivors negatively affects the ability to return to work, social participation, and quality of life^11,12^. In line with this, and as recognized in recent works, there is an unmet need of increased understanding of the biological processes influencing post-stroke cognitive outcomes^13,14^. Indeed, post-stroke cognitive outcome can be conceptualized as a summation of direct stroke-related effects, such as infarct size and localization, but also pre-stroke cognitive reserve, concomitant microvascular and neurodegenerative pathologies, such as cerebral small vessel disease and (early) Alzheimer’s disease, with the molecular interplay poorly understood^13–15^. Identification of protein biomarkers associated with post-stroke cognitive function has the potential to indicate candidate pathophysiological processes, inform further mechanistic investigations, and ultimately contribute to the identification of novel therapeutic targets. Blood-based biomarkers also have the potential to guide interventions for persons with increased risk for cognitive decline after stroke.

Recently, our group reported multiple novel candidate plasma protein biomarkers of neurological outcome after ischemic stroke by measuring 91 proteins involved in neurobiological processes (Olink Neurology Panel)^16^. The same assay has been used by others to identify novel plasma protein biomarkers of Alzheimer’s disease (AD)^17–19^ and cognitive function in population-based cohorts^20^, but to the best of our knowledge not in stroke cohorts. Given that post-stroke cognition is likely influenced by both stroke-specific and general neurodegenerative processes^13,14^, blood-based biomarkers of cognitive function are not necessarily completely overlapping between post-stroke and population-based cohorts. We thus sought to profile these proteins for association with cognitive outcome in a relatively young ischemic stroke cohort with long-term follow-up. Our primary aim was to identify plasma protein biomarkers of long-term cognitive outcomes in the early phase after ischemic stroke, that in turn can point towards biological processes in merit of further investigation.

## Methods

### Study population and baseline characteristics

This study sample comprises of 205 cases from a sub-study within the longitudinal observational cohort study *Sahlgrenska Academy Study on Ischemic Stroke* (*SAHLSIS*) with available blood samples from all sampling points during follow-up, i.e. from the acute phase, and follow-up visits at 3 months and 7 years, and data from cognitive testing at long-term follow-up. The design of *SAHLSIS* and the present sub-study has been described in detail elsewhere^16^. In short, cases with first-ever or recurrent acute ischemic stroke at age 18–69 years were included consecutively at the stroke unit at the Sahlgrenska University Hospital, Gothenburg in 1998 to 2003. Acute ischemic stroke was defined as an episode of focal brain dysfunction with acute onset, lasting > 24 h, and of presumed vascular cause with no signs of hemorrhage or another cause on neuroimaging. All patients included in this study underwent imaging of the brain (computed tomography and/or magnetic resonance) as part of the clinical routine investigation. Participants underwent additional work-up according to national guidelines and were excluded if further evaluation showed another etiology than stroke. Complete information on vascular risk factors was registered at the 3-month follow-up by examinations and a structured questionnaire. Definitions were as follows, hypertension: pharmacological treatment for hypertension and/or systolic blood pressure ≥ 160 mmHg, and/or diastolic blood pressure ≥ 90 mmHg; diabetes mellitus: dietary or pharmacological treatment and/or fasting plasma glucose ≥ 7.0 mmol/L; hyperlipidemia: pharmacological treatment, total fasting serum cholesterol > 5.0 mmol/L, and/or LDL > 3.0 mmol/L; smoking: current smoking as opposed to never or former (cessation at least one year from inclusion). Information on attained education was obtained from questionnaires and grouped into categories: (1) elementary school or less (completed or non-completed elementary school), (2) upper secondary school or vocational school, and (3) university. Acute stroke severity was defined as the worst score by the Scandinavian Stroke Scale (SSS) within the first 7 days after stroke onset and converted to the internationally more commonly used NIH Stroke Scale (NIHSS) scores using an algorithm^21^ to facilitate comparisons with other studies. Of note, during the inclusion period of this study, acute revascularization therapy was not yet part of the routine clinical practice.

### Cognitive outcome 7 years post-stroke

All surviving study participants in the *SAHLSIS* sub-study described above were invited to follow-up visits approximately 7 years after the index stroke (median 7.5 years, IQR − 5.9 to + 5.6 weeks), i.e. these visits took place from 2006 to 2011. The participation rate was 82%, and reasons for drop-out have been described in detail elsewhere^22^. Cognitive testing was performed by the Barrow Neurological Institute Screen (BNIS) for Higher Cerebral Functions, a comprehensive and reliable screening instrument for cognitive dysfunction^23^ with high sensitivity^22,24^ and good validity^24^ in stroke patients. All study participants were tested by the same research nurse (Ingrid Eriksson), who was trained in administering the BNIS and continually supervised by two neuropsychologists (HS and Caisa Hofgren). The BNIS total score (maximum 50) reflects the overall cognitive function and consists of a pre-screen (level of consciousness 3 points, basic language 3 points, and level of cooperation 3 points) to evaluate whether the participant is capable to take part in further testing, and 7 ensuing subscales: speech and language functions (15 points), orientation (3 points), attention/concentration (3 points), visuospatial function and visual problem solving (8 points), memory (7 points), affect (4 points), and awareness (1 point). The BNIS is intended as a screening test to identify persons with possible cognitive dysfunction that might benefit from further neuropsychological evaluation, and inherently a higher sensitivity than specificity is thus preferred. However, there is yet no established optimal cut-off value for indicating possible cognitive dysfunction by the BNIS and different cut-offs have been suggested^23^. An early suggestion was < 47 points for persons ≤ 55 years and < 43 points for persons > 55 years of age^25^. The cut-off level for persons ≤ 55 years of age was supported in a later validation study of the BNIS based on conventional neuropsychological tests in a cohort of stroke cases with good functional outcome, but for cases > 55 years of age the optimal cut-off level was < 41 points (sensitivity 92% and specificity 85%)^24^. For descriptive purposes in the present study, the cut-off values for possible cognitive dysfunction that we used thus were < 47 points for cases ≤ 55 years and < 41 points for cases > 55 years.

### Other assessments at the 7-year follow-up visit

Presence of vascular risk factors was reassessed by questionnaires. Recurrent strokes and neurological comorbidities with plausible influence on protein measurements or outcome were identified through questionnaires and national registers, and diagnoses were verified by review of medical records as described^26^.

### Blood sampling

Venous blood samples were drawn during the acute (median 4 days after index stroke, interquartile range (IQR) 3–6 days) and convalescent (3 months; median 98 days, IQR 93–109 days) phases. Additional blood samples were drawn at the 7-year visit. On all occasions, blood was collected between 8.30 and 10.30 a.m. after an overnight fast. Blood was drawn in tubes containing 10% by volume ethylenediaminetetraacetic acid (EDTA). Plasma was isolated within 2 h by centrifugation at 2000×g at 4 °C for 20 min, aliquoted, and stored at − 80 °C pending analysis.

### Measurement of proteins levels

Plasma levels of 91 neurology-related proteins were measured using the highly sensitive and specific Proximity Extension Assay (PEA)^27^ using the Neurology panel (Olink Proteomics, Uppsala, Sweden). In brief, analyses were performed according to the manufacturer’s protocol by a board-certified laboratory technician at Olink, blinded to the clinical information. The 3 samples from each case were placed on the same plate, to limit the impact of inter-run variability. Samples not passing quality control (*n* = 5) were excluded from further analysis. Plasma protein levels were measured as Normalized Protein eXpression (NPX) values, which is a relative quantification given on a log2 scale. For individual protein read-outs below the limit of detection (*n* = 19 in total), levels were set to the lowest limit of detection (see Table S1 for details). Mean intra- and inter-run coefficients of variation was 6.5% and 11.5%, respectively, as detailed^16^. All investigated proteins, including their full protein name, Uniprot ID and encoding gene, are listed in Table S1. Of note the version of the Olink Neurology panel used here did not contain the neuroaxonal damage marker Neurofilament light chain (NfL). However, we have previously measured serum NfL concentrations using a Single Molecule Array (SiMoA) assay in the *SAHLSIS*, and reported these results in relation to functional and neurological outcomes^26^. Thus, we included these NfL data in the present study to be able to compare the results for the other proteins with NfL.

### Statistical analyses

Bivariate correlations were assessed by Spearman’s rank correlation. Proteins were clustered hierarchically based on degree of covariation according to Euclidean distance. The total BNIS score was treated as a continuous variable and linear regression was used to investigate associations between individual protein levels and BNIS scores. Scatter plots of all assessed proteins by BNIS scores were manually viewed, and 3 deemed outliers were removed from the linear regression analyses (beta-NGF: 1 study participant, all sampling points; CLM-6; 1 study participant, all sampling points; GZMA: 1 study participant, acute phase levels). For plasma proteins measured by the Olink PEA the NPX value is given on a log2 scale, and thus the yielded β-value corresponds to the predicted change in BNIS score per each doubling of the protein level. Previously measured serum NfL levels (pg/mL) were therefore log2 transformed to harmonize these β-values with the Olink data.

Pre-specified adjustments were made for the potential confounding factors age at inclusion, sex, educational level, and day of blood draw (model 1), and additionally also for acute stroke severity (model 2). Our primary aim was to identify candidate acute and 3-month markers of long-term cognitive outcome independent of these clinical variables, but we also performed univariable and cross-sectional analyses of protein levels and cognitive outcome at the 7-year follow-up for comparisons.

Study participants with missing information were as follows: education *n* = 4; hyperlipidemia at baseline: *n* = 4; information on vascular risk factors at the 7-year follow-up: *n* = 2; time until blood draw: *n* = 3 (acute phase), *n* = 1 (3 months); missing Olink protein measurements due to samples not passing quality control: *n* = 2 (acute phase), *n* = 2 (3 months), *n* = 1 (7 years); missing serum NfL levels: *n* = 45 (acute phase), *n* = 5 (3 months), *n* = 3 (7 years). Participants with missing information were only excluded in statistical analyses including this (these) specific variable(s). From 1998 to 2000, serum was not collected in the acute phase, explaining the majority (*n* = 44) of the missing NfL measurements at this time-point.

Least absolute shrinkage and selection operator (LASSO) regression was used to identify proteins at each sampling point, that contributed to the prediction of cognitive outcome above the clinical variables age, sex, education, sampling day (multiprotein model 1) and stroke severity (multiprotein model 2). Repeated cross-validation (fivefold, repeated 20 times) and a grid search approach with a fixed alpha of 1 (R package glmnet) were used to identify the optimal lambda value for each model. For acute phase protein levels, two sets of LASSO regression procedures were made: one including acute NfL levels (*n* = 45 participants with missing information thus excluded) and one without this variable. As acute NfL levels were not among selected proteins in the first set, we proceeded with the second set only.

Linear regression analyses for selected proteins were rerun in a sensitivity analysis excluding participants with a recurrent stroke between inclusion and the 7-year follow-up and/or a neurological comorbidity (*n* = 29).

For the individual protein analyses, the Benjamini and Hochberg method was used to control the false discovery rate (FDR) of multiple testing^28^. Associations with FDR < 0.05 were considered statistically significant, and associations with FDR ≥ 0.05 but a two-tailed *p* < 0.05 were considered suggestive. However, it is of note that adjustment for multiple comparisons by number of investigated proteins (*n* = 92) is arbitrary as many of the investigated proteins were highly correlated (Tables S2–S4), thus not equating to 92 independent comparisons. In the remaining analyses, a two-tailed *p* < 0.05 was considered statistically significant.

Statistical analyses were performed in R version 4.4.1 or IBM SPSS Statistics version 29.

## Results

Of the 209 participants with measured plasma protein levels, 205 completed the 7-year BNIS test and were thus included in the present study. Reasons for not completing the BNIS were aphasia (*n* = 2) and poor vision (*n* = 2). For the included study participants, clinical characteristics at baseline with correlations to the total BNIS scores at the 7-year follow-up are given in Table 1. Most participants experienced a mild stroke, and none received acute recanalization therapy (intravenous thrombolysis or thrombectomy). Total BNIS scores were nearly normally distributed (median 41, IQR 37–44) and 121 participants (59%) had a score indicative of possible cognitive dysfunction. Age and educational level were moderately correlated to 7-year BNIS scores (*r* = − 0.36, *p* < 1 × 10^−6^ and *r* = 0.36, *p* < 1 × 10^−6^, respectively), whereas hypertension, diabetes mellitus, and stroke severity (NIHSS) were weakly so (|r| < 0.3, Table 1).

**Table 1:** **Clinical characteristics and Spearman correlations to cognitive function (total BNIS scores) 7 years after index ischemic stroke.**

	Study participants; *n* = 205 ischemic stroke cases	Correlations to BNIS scores
**Baseline**		
Age, years, median (IQR)	55 (49–61)	− 0.36***
Male, n (%)	133 (65)	− 0.16*
Education		
Elementary school or less, n (%)	55 (27)	0.36***
Upper secondary school or vocational school, n (%)	85 (42)	
University, n (%)	61 (30)	
Hypertension, n (%)	110 (54)	− 0.15*
Diabetes mellitus, n (%)	30 (15)	− 0.22**
Smoking, n (%)	71 (35)	− 0.02
Hyperlipidaemia, n (%)	147 (73)	− 0.06
Acute stroke severity, NIHSS score, median (IQR)	2.5 (1.2–4.6)	− 0.26***

*IQR* interquartile range, *NIHSS* National Institutes of Health Stroke Scale, *BNIS* Barrow Neurological Institute Screen for Higher Cerebral Functions.

*p < 0.05, **p < 0.01, ***p < 0.001.

### Associations between acute-phase protein levels and 7-year cognitive outcome

**Fig. 1:**
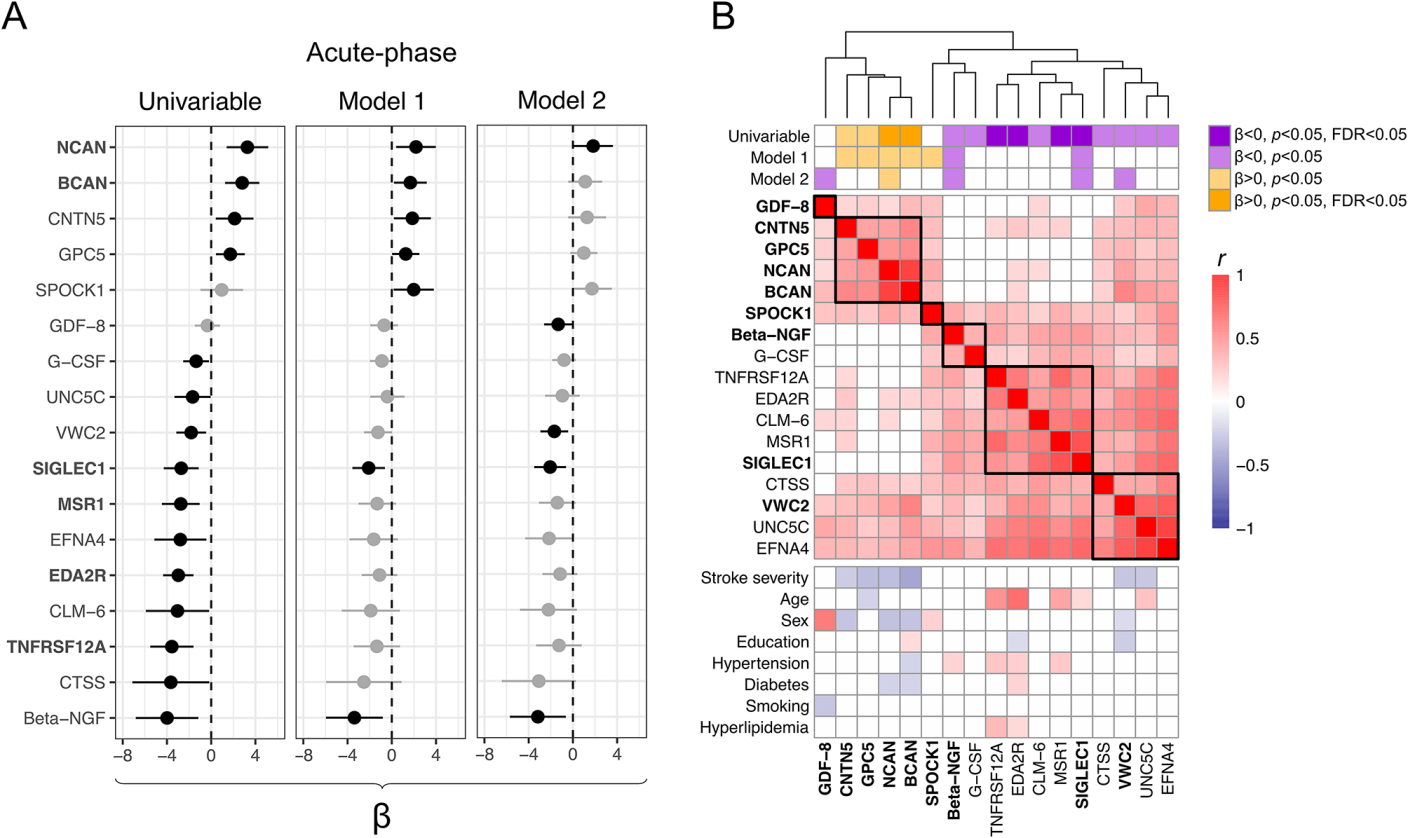
**Associations between individual protein levels in the acute phase of ischemic stroke and 7-year cognitive outcome, together with correlations for 17 selected proteins.** (**A**) Associations between protein levels in the acute stroke phase and cognitive outcome 7 years after index stroke for proteins associated at *p* < 0.05 in any analysis of individual protein levels. Associations are visualized in forest plots with β values and 95% confidence intervals derived from univariable linear regression, and multivariable linear regression models adjusted for age, sex, education and blood sampling day (model 1). Associations in model 2 are additionally adjusted for acute stroke severity (NIH stroke scale [NIHSS]). Associations with *p* < 0.05 are shown in black, and for proteins marked in bold text font, univariable associations were associated at false discovery rate (FDR) < 0.05. The β-value corresponds to the predicted change in the Barrow Neurological Institute Screen (BNIS) for Higher Cerebral Functions total score per each doubling of protein level and a positive β-value to higher protein levels being associated with a more favorable cognitive outcome. (**B**) Hierarchical protein clusters in the acute stroke phase for proteins associated with cognitive outcome at *p* < 0.05 in any analysis of individual protein levels. The full hierarchical cluster tree based on degree of covariation according to Euclidean distance is displayed and a selected number of clusters are visually highlighted by bold squares in the heat map. Information on the association between protein levels and cognitive outcome at 7 years according to the BNIS total score are given in the first rows, with adjustments as in Panel A. Proteins selected by LASSO regression to contribute with information in multiprotein models of 7-year cognitive outcome are marked in bold. In following rows, bivariate Spearman’s rank correlation coefficients (r) between protein levels and clinical variables are displayed, where correlations with *p* < 0.05 are marked in colour. Positive correlations (*r* > 0) between protein levels, increasing acute stroke severity (NIHSS), and age are marked red, and inverse correlations (*r* < 0) are marked blue. For sex, a positive correlation (*r* > 0, red boxes) corresponds to higher protein levels in males than females, and for remaining clinical variables a positive correlation corresponds to higher protein levels in participants with that factor prevalent.

Results from the linear regression analyses for associations between individual protein levels measured in the acute phase and 7-year cognitive testing results are shown in Fig. 1 and detailed in Table S5. In the univariable analyses acute phase levels of 6 proteins were significantly associated with 7-year BNIS scores (FDR < 0.05, Fig. 1A). Of these, BCAN, NCAN and SIGLEC1 were also suggestively associated (*p* < 0.05) after adjustment for age, sex, educational level, and day of blood draw (model 1), with NCAN and SIGLEC1 remaining so after additional adjustment for acute stroke severity (model 2). Acute phase levels of 6 further proteins were suggestively associated with BNIS scores in model 1 and/or model 2 (*p* < 0.05, Fig. 1A), but none with FDR < 0.05.

Among the 17 acute-phase proteins associated with cognitive outcome at *p* < 0.05 in any of the regression analyses, there were 6 main protein clusters. Higher protein levels were associated with lower cognitive performance in 4 clusters, and with better cognitive performance in 2 clusters (Fig. 1B).

We next applied LASSO regression to identify the proteins contributing with information in acute phase multiprotein models of cognitive outcome. Starting with all 92 proteins, 9 proteins across the 6 main clusters were selected (Table 2). In the first model that included information on age, sex, education, and sampling day (multiprotein model 1), 8 proteins were selected, and in the second model that also included stroke severity (NIHSS), 7 proteins were selected.

**Table 2:** **Proteins selected by LASSO regression to contribute with information in multiprotein models of 7-year cognitive outcome.**

Order	Acute-phase		3-month		7-year	
	Model 1	Model 2	Model 1	Model 2	Model 1	Model 2
1	Beta-NGF	Beta-NGF	**GFR-alpha-1**	**GFR-alpha-1**	**NCAN**	**SIGLEC1**
2	SPOCK1	SPOCK1	BCAN	CLEC10A	**SIGLEC1**	CDH3
3	**SIGLEC1**	**SIGLEC1**	SIGLEC1	BCAN	**VWC2**	**NCAN**
4	**NCAN**	**NCAN**	**NCAN**	NMNAT1	**ADAM 22**	**ADAM 22**
5	VWC2	VWC2	NMNAT1	HAGH	–	**VWC2**
6	GPC5	GDF-8	HAGH	Beta-NGF	–	–
7	CNTN5	GPC5	**NfL**	SIGLEC1	–	–
8	**BCAN**	–	Beta-NGF	–	–	–
9	–	–	CD200R1	–	–	–
10	–	–	**VWC2**	–	–	–

Proteins are listed by order of importance. Bold indicates proteins associated to cognitive outcome below the statistical significance threshold FDR < 0.05 in univariable analyses. Model 1 includes listed proteins together with covariates age, sex and educational level. Model 2 includes variables as in model 1 together with acute stroke severity (NIHSS). Models including acute-phase and 3-month protein levels were additionally adjusted for sampling day.

### Associations between convalescent phase protein levels and 7-year cognitive outcome

**Fig. 2:**
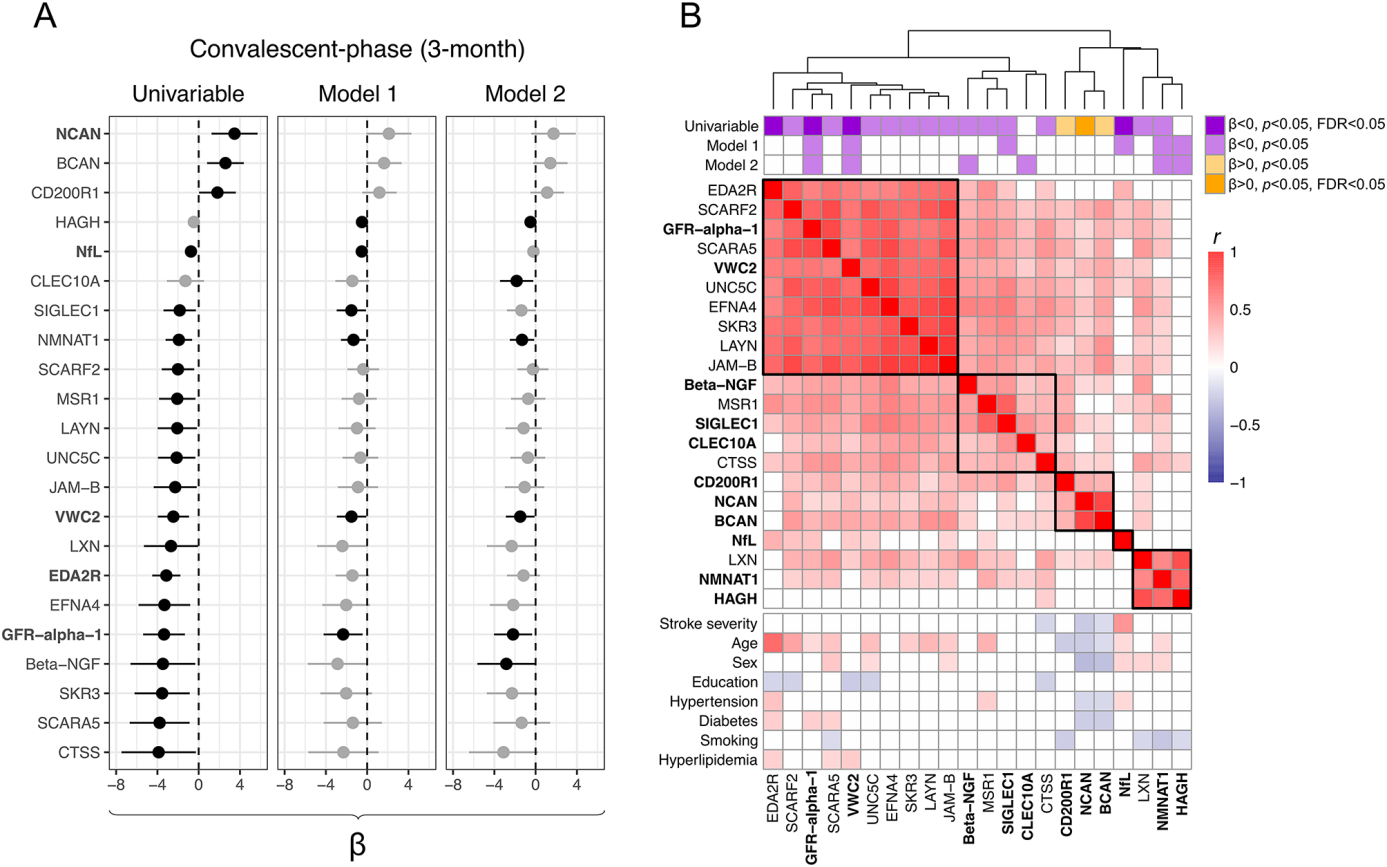
**Associations between individual protein levels in the convalescent stroke phase and 7-year cognitive outcome, together with correlations for 22 selected proteins.** Associations between protein levels at the 3-month follow-up and cognitive outcome 7 years after index stroke, and hierarchical clusters for proteins associated with cognitive outcome at *p* < 0.05 in any analysis of individual protein levels. Panels (**A**,**B**) as in Fig. 1.

At the 3-month follow-up, individual levels of 22 proteins were associated with 7-year cognitive outcome at *p* < 0.05 in either univariable or multivariable analyses, of which 5 proteins were significant at FDR < 0.05 in the univariable analyses (Fig. 2A and Table S5). These 22 proteins clustered into 5 main groups (Fig. 2B), where only the cluster of BCAN, NCAN and CD200R1 contained proteins for which higher 3-month levels were associated with better cognitive performance; for the remaining clusters inverse associations were observed. Ten proteins (including BCAN, NCAN, beta-NGF, SIGLEC1 and VWC2) were common to both the acute and convalescent stroke phase, but there were also differing examples. For instance, and in contrast to acute phase levels, 3-month levels of SPOCK1 and GDF-8 were not associated to cognitive outcome (*p* > 0.42 for both, Table S5). Also, levels of NfL, HAGH, and NMNAT1 were associated when measured at 3 months, but not in the acute stroke phase (*p* > 0.13 for all, acute phase levels, Table S5). Further, 3-month levels of NfL were only associated to 7-year BNIS scores before adjustment for acute stroke severity (i.e. univariably and in model 1, Fig. 2A, and Table S5).

Three-month levels of 11 proteins were selected using LASSO regression to contribute with information in multi-protein models of cognitive outcome and these included both common and differential proteins compared to the acute phase sampling (Table 2).

### Cross-sectional associations between protein levels and cognitive function 7 years post-stroke

**Fig. 3:**
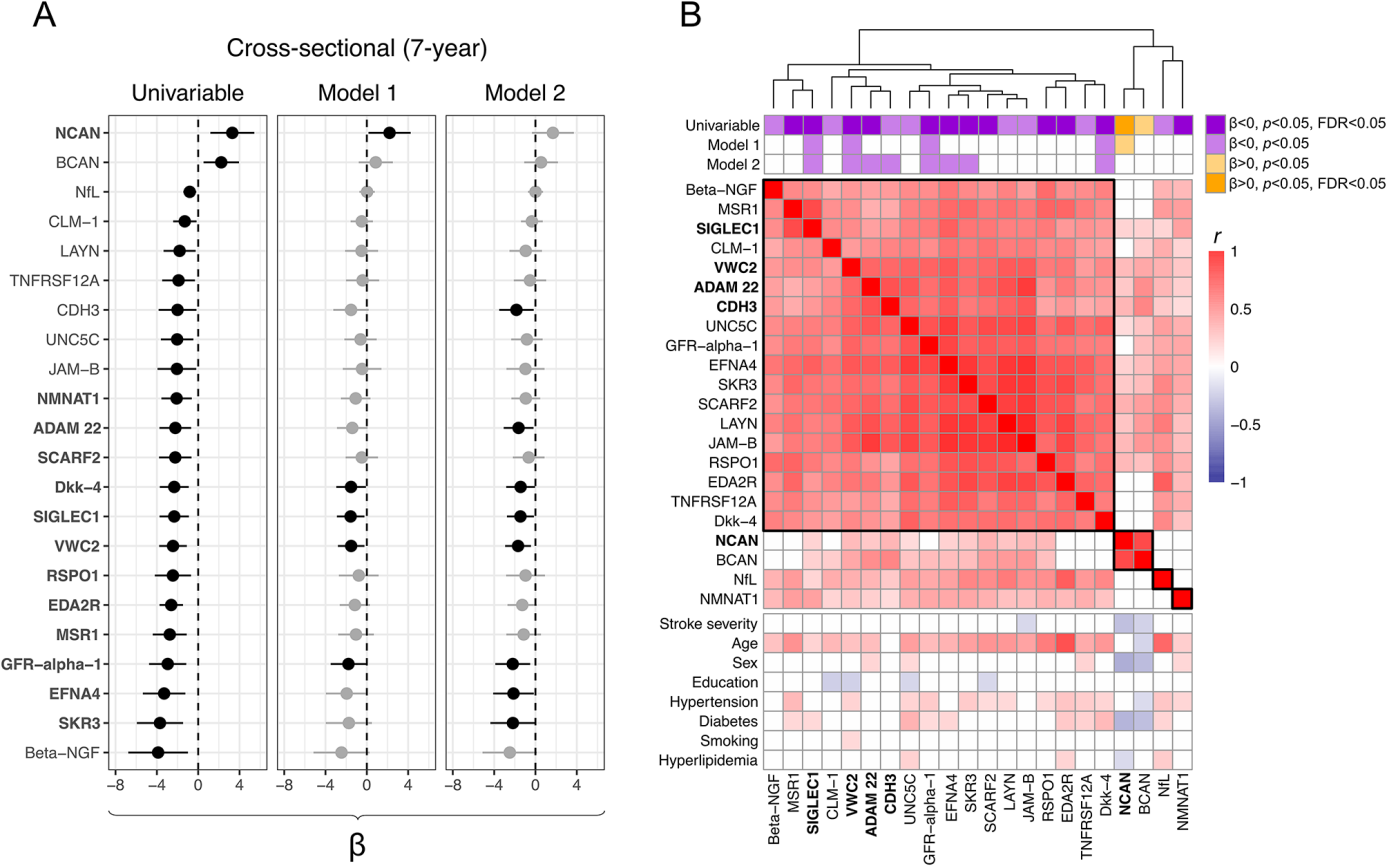
**Cross-sectional associations between individual protein levels and cognitive outcome at the 7-year follow-up, together with correlations for 22 selected proteins. **Cross-sectional associations between protein levels and cognitive outcome at follow-up 7 years after index stroke, and hierarchical clusters for proteins associated with cognitive testing scores at *p* < 0.05 in any analysis of individual protein levels. Panels (**A**,**B**) as in Fig. 1, except linear regressions models were not adjusted for sampling day.

For comparison, we also investigated cross-sectional associations between protein levels and cognitive testing scores at the 7-year follow-up (Fig. 3A and Table S5). There were fewer clusters of proteins associated to concomitant BNIS scores (Fig. 3B, and Table S5), and only 5 proteins contributed with information in multiprotein models (Table 2). Of these, all but ADAM22 and CDH3 had been associated to cognitive outcome also when measured at an earlier sampling point.

### Sensitivity analyses

For proteins associated to 7-year BNIS scores at *p* < 0.1 in the adjusted analyses of individual protein levels (model 1–2), linear regression analyses were rerun in a sensitivity analysis excluding cases with recurrent stroke and/or a neurological comorbidity before the 7-year follow-up (*n* = 29), with results essentially unchanged (Table S6).

## Discussion

This exploratory study of 92 neurology-related proteins is the broadest investigation of putative blood protein biomarkers for long-term post-stroke cognitive outcome to our knowledge. We identified novel candidate biomarkers that followed different trajectories after the acute stroke phase, clustered into separate groups, and contributed with information in multi-protein models of 7-year cognitive outcome. We interpret these results as a reflection in blood of different biological processes involved in post-stroke cognition.

Of the candidate protein biomarkers reported in this study, NfL has been previously investigated in blood for association to post-stroke cognitive outcomes. Our present finding that higher 3-month NfL concentrations are associated with lower cognitive function in young and middle-aged ischemic stroke survivors with mild stroke is in line with the reported associations to post-stroke cognitive decline and dementia risk in stroke cohorts of older age^29,30^. NfL concentrations have also been associated with stroke severity^26^, infarct size^31^, cerebral small vessel disease burden^29,32^, and post-stroke functional outcome^26,31,33^, factors in turn known to influence and/or correlate with post-stroke cognitive function. For many of the other markers we report here, previous investigations suggest a role in biological processes of importance for cognitive function, e.g. in population-based cohorts^20,34,35^, in relation to vascular dementia^36^, AD^17–19,37,38^, and cerebral white matter hyperintensities^39^, as well in experimental work^40,41^. These candidate markers include BCAN and NCAN that are emerging biomarkers of cognitive and neuroimaging phenotypes^17,20,35,42^, and GFR-alpha 1 that has been associated with incident dementia^43^.

Our investigation complements previous studies with information on the profiled proteins in relation to cognition specifically in the post-stroke setting. It is conceivable that cognitive function post-stroke is influenced by both infarct-related and general neurodegenerative processes, and it follows that individual biomarkers may reflect both or either of these processes. Possibly reflecting an infarct-related process, we note that some proteins (e.g. NfL, BCAN, CLEC10A, CNTN5, GDF-8, GPC5, HAGH, and SPOCK1) were associated to cognitive outcome when measured in the acute or convalescent stroke phase, but not in the adjusted cross-sectional analyses at the 7-year follow-up in our study. Further investigations are of course needed to confirm if this represents true stroke-specific trajectories, but we have previously reported differences in protein levels by time after stroke in the same study cohort^16,26^. For the neuroaxonal injury-biomarker NfL, the observed association to cognitive outcome is likely explained by reflection of stroke severity, of which subacute NfL levels are especially informative^26,31,33^. In line with this, the association between 3-month NfL levels and cognitive outcome was not retained after adjustment for acute stroke severity in the present study. In contrast, levels of all remaining examples above except CNTN5, contributed in multi-protein models also including acute stroke severity, suggesting that there is a time-window early post-stroke where plasma levels of some proteins reflect biological processes that influence long-term cognitive outcome independent of stroke severity. Validation in additional stroke cohorts is needed to confirm these findings.

**Fig. 4:**
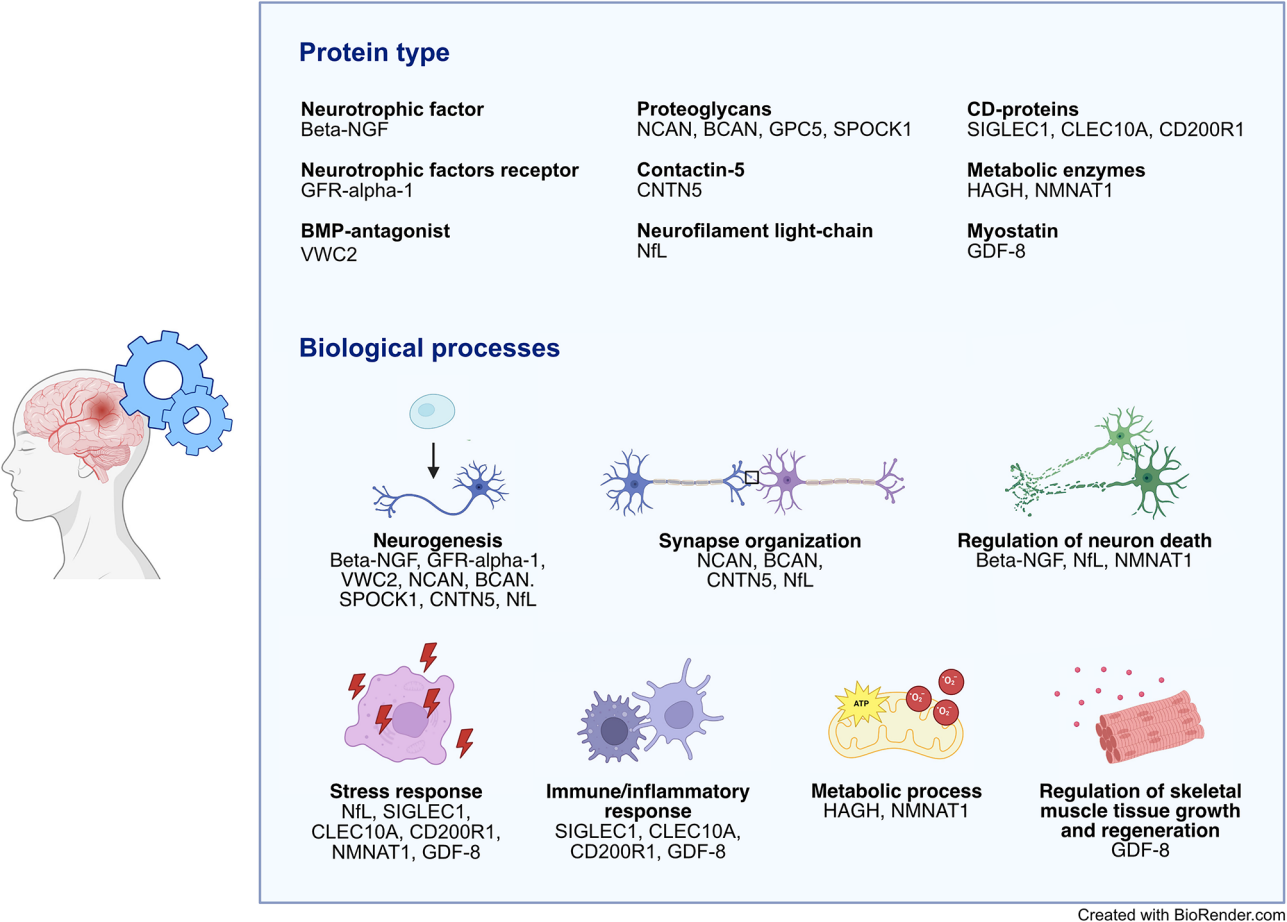
**Candidate acute and/or 3-month markers of long-term cognitive outcome. **Protein types and selected gene ontology (GO) biological processes for the proteins associated at *p* < 0.05 in the adjusted analyses of individual proteins levels (model 1 and/or 2) or contributing with information in multiprotein models of long-term cognitive outcome, in the acute or convalescent stroke phase. *BMP-antagonist* bone morphogenetic protein-antagonist, *CD-proteins* cluster of differentiation proteins.

Our goal was to identify candidate protein biomarkers in the early post-stroke phase of long-term cognitive outcome, that in turn can point towards biological processes in merit of further investigation. In our data, we observed the highest number of clusters among associated proteins in the acute and convalescent stroke phase, and it follows that a higher number of proteins contributed with information in multi-protein models at these sampling points compared to the cross-sectional 7-year analyses. This likely means that a larger number of biological processes influencing cognitive outcome was reflected early after stroke by profiling of the proteins investigated in this study. Further, it was possible to discern different protein types and functions among the candidate acute and 3-month markers (Fig. 4). For instance, BCAN and NCAN are brain-specific extracellular matrix chondroitin sulfate proteoglycans, whereas GDF-8 (myostatin) and NfL both clustered singly among candidates. Metabolic enzymes NMNAT1 and HAGH (also known as glyoxalase II) clustered together, both involved in cellular processes of importance in neurodegenerative diseases and ageing, including oxidative stress protection^44,45^. NMNAT1 is protective after experimental ischemic stroke^46–48^ and increased plasma levels of HAGH has been reported in pre-symptomatic AD^18^. SIGLEC1 and CLEC10A are both immune cell-specific membrane proteins differentially expressed in microglia during AD^49^, and differential expression of *SIGLEC1* in blood leukocytes is associated with progression of cerebral white matter hyperintensities^39^. VWC2, a bone morphogenetic protein (BMP)-antagonist predominantly expressed in the brain (GTEx) clustered together with the neurotrophic factor receptor GFR-alpha-1, and both have previous support as potential novel cognition biomarkers^17,19,20,43^. VWC2 and GFR-alpha-1 clustered together with additional proteins including the endothelial BMP-receptor SKR3 (i.e. ACVRL1 or ALK1) which plays a central role in vasculogenesis^50^, and both GFR-alpha 1 and SKR3 have been suggested as a possible mediators between poor cardiovascular health and incident dementia^43^. Taken together, proteins with currently annotated metabolic, neuroprotective, neurotrophic, immunologic, and angiogenic functions are among the identified novel candidates, pointing to a role of such pathways in post-stroke cognitive function.

Strengths of this study include the inclusion of consecutive and clinically well-characterized study participants followed long term. We used a validated, sensitive cognitive screening instrument spanning across different cognitive domains^23^, and analyzed protein level associations to the entire cognitive testing scale score. Repeated blood samplings were standardized, and levels of a large number of proteins were analyzed simultaneously by a multiplex method with high specificity^27^, with undertaken measures to limit the impact of run-to-run variability as detailed^16^. We have also previously provided in-depth information on protein levels trajectories after stroke, differences between stroke cases and controls, and associations to stroke severity and neurological outcome for the proteins included in the present study^16,26^, enabling comparisons.

There are also important study limitations to consider, including previously discussed^16^ impact on generalizability to cases with more severe strokes and older age, and circulating levels not necessarily reflecting protein levels in the cerebrospinal fluid (CSF) or brain. Indeed, correlations between plasma and CSF levels have been reported to vary among the proteins investigated^17^. Also, this study is exploratory, and the results need replication. However, we know of no similar cohort for validation at this time, and a majority of the reported proteins have previous support as candidate cognition biomarkers as detailed above. Moreover, cognitive function was only evaluated at the long-term follow-up, and we did not have information on cognitive ability pre-stroke or cognitive testing scores in age and sex-matched controls, precluding discernment of direct stroke-caused impairments and early versus late post-stroke changes in cognitive ability. Still, a majority of the relatively young study participants investigated here was in full-time employment at the time of index stroke, indicating a normal cognitive functioning at baseline, and efforts to provide in-depth data on trajectories of both biomarkers and cognitive outcome after stroke are underway by both us and others^13,51,52^. Also, levels of NfL and the other 91 investigated proteins were measured using two different methods (i.e. NfL with the quantitative SiMoA technology and the other proteins with the Olink Proximity Extension Assay) and in serum and plasma, respectively, why effect sizes between NfL and the other protein levels cannot be directly compared. Further, the inclusion period of the study was relatively long, and plasma samples were stored several years at − 80 °C prior to analysis. However, we visually inspected scatter plots of acute plasma proteins levels (the samples stored the longest) by sampling date and found no instances of lower protein levels in participants sampled early during the study period, i.e. we found no signs of plasma protein degradation by storage time. Finally, to investigate possible associations to infarct volume and cerebral small vessel disease, an interesting future perspective is to complement our results with neuroimaging data, and to investigate possible differences between ischemic stroke etiological subtypes that the present study is underpowered to uncover.

Here, we conducted an exploratory study of 92 neurology-related circulating proteins and identified candidate novel blood biomarkers that likely reflect different biological processes of importance for long-term cognitive function after stroke. Validation in independent stroke cohorts and additional mechanistic investigations are warranted.

## Electronic supplementary material

Below is the link to the electronic supplementary material.

Supplementary Material 1

## Data Availability

Summary statistics are provided as Supplemental material. Anonymized data will be shared upon reasonable request from a qualified academic investigator to the corresponding author as long as data transfer agrees with EU legislation on the general data protection regulation (GDPR) and with decisions by the Ethical Review Board of Sweden and the University of Gothenburg, the latter which should be regulated in a data transfer agreement.
